# College English Reading Teaching Integrating Production Oriented Approach from the Perspective of Artificial Intelligence

**DOI:** 10.1155/2022/3411203

**Published:** 2022-06-24

**Authors:** Xi Wang, Ke Yan

**Affiliations:** ^1^School of Foreign Languages and International Education, Chengdu Technological University, Chengdu 611730, Sichuan, China; ^2^Department of Public Teaching, Nanyang Medical College, Nanyang 473000, Henan, China

## Abstract

The objectives are to solve many problems in traditional English reading teaching, such as the passive acceptance of students' learning situation, the rigid teaching mode of teachers and the difficulty in taking into account the individual needs of each student, and the forced averaging of students' English learning ability and level. Firstly, a detection method of English reading level based on the Backpropagation neural network (BPNN) is designed. Secondly, the teaching process and specific teaching plan are designed with the Production Oriented Approach (POA). Finally, the detection method of English reading level is verified by simulation experiments, and the effect of the teaching method integrating POA is analyzed. The results show that (1) when the learning rate is 0.26 and the training time is 1000 times, the mean square error (MSE) of the model is the smallest. (2) The results of the practical experiment indicate that the number of people who sign up for the college English proficiency test, the number of actual exams, and the pass rate of the test in the experimental group using the teaching method are higher than those in the control group. Moreover, the number of people in the experimental group increased more than that in the control group after the experiment. There is no significant difference in the test results of the two groups before the experiment, and the average test scores of the experimental group are obviously higher than those of the control group after the experiment. The *p* value of the two groups of results is less than 0.05 in the *t*-test, indicating that there is a significant difference in the English reading level between the two groups after the experiment. Simultaneously, the difference between the results of class A before and after the experiment is larger than that of class B, showing that the teaching method integrating POA is more effective for students with higher English levels. Therefore, compared with the traditional teaching method, the teaching mode integrating POA is more effective in improving the English reading level of college students. It aims to transform POA theory into practice and provide a great reference for college English reading teaching.

## 1. Introduction

The requirements of college English teaching courses emphasize that on the basis of developing students' comprehensive language ability, they should focus on improving students' ability to acquire information, process information, analyze problems and solve problems in English [1]. In the current era of mature wireless networks and abundant electronic resources, new teaching theories and methods that conform to the characteristics of the times have been gradually put forward. The concept teaching method advocates that teaching starts from concepts and allows students to discover the differences between different language systems through conscious comparative analysis [2]. The task-based teaching method is based on system functional linguistics and organizes teaching by designing tasks, emphasizing “learning by doing” [3]. The presentation, assimilation, and discussion (PAD) teaching method retains the lecture style teaching method and absorbs the core concept of cooperative learning, which increases the proportion of discussion in the classroom [4]. The Production Oriented Approach (POA) is an effective way to solve the problem of separation of theory and practice in the process of teaching.

Zhou pointed out that the POA is a teaching theory based on the output-driven hypothesis. In the whole teaching process, the teacher is the center, promoting the development and implementation of the whole teaching activities. By adopting the POA in the teaching process, teachers can reconstruct traditional classroom teaching, better solve the problems existing in traditional college English courses, and further implement a student-centered teaching model [5]. Wang proposed the POA theory of combining learning and application in foreign language teaching in view of the current situation of separation of learning and application in foreign language teaching in China. The theory takes the unit task with potential communicative value as the teaching goal. Under the guidance of the teacher, the students selectively learn the input materials and finally achieve the effective output and complete the communicative task. It was found through research that this method can effectively stimulate students' enthusiasm for learning and creative willingness [6]. Zhang proposed that the POA with effective learning as the core was put forward to address the problems of text centralization, separation of learning and use, and “dumb English” in Chinese English classroom teaching. This study applied POA to college English classroom teaching to examine its impact on students' English learning and to explore its implications for English teaching [7]. Xie surveyed 172 students from English majors and non-English majors to investigate their needs for POA and their perceptions of strategies for the effective implementation of a business English program at a university in China. The research results indicated that the application of POA theory was of great significance for the reform and theoretical development of business English teaching in Chinese universities and for further innovation and improvement of the teaching process in other cultural and educational contexts [8].

With the breakthrough and development of wireless networks and artificial intelligence (AI), rich extracurricular teaching resources have optimized the quality of classroom reading materials, and the combination of offline and online teaching methods has broken through the limitations of time and space and improved learning efficiency. AI technology can track and record the trajectory of college students' English learning and use new technologies such as data mining, regression prediction, and correlation analysis to generate an English learning database, summarize the laws of college students' English learning, and analyze the personalized learning characteristics of each student [9]. In the process of the development of POA theory, the theoretical assumptions for college English teaching problems are applied to teaching practice, which is not only expected to improve the effect of college English teaching but also can help the development of the theory.

## 2. Methods

### 2.1. POA

POA is a new foreign language teaching theory constructed by Chinese scholars. The purpose is to solve the problem of “separation of learning and application” in college English. It has undergone several revisions and has been perfected by the cycle of theory-practice-interpretation [10]. The teaching operation process of this system is based on the original input and output theory and advocates the division of labor and cooperation between teachers and students in English learning [11]. The related research on POA mainly focuses on college students, among which the undergraduates as the research object account for about 88% of the available research literature, which is in line with the requirements of the theory for middle-level and high-level learners [12]. The POA theory creation team of the International Language and Education Research Center of Beijing Foreign Studies University, China, has carried out a lot of teaching practice in major universities in China and has achieved fruitful research results. The textbook compilation mode of “two-wheel drive of teaching theory-action research” is proposed. The basic idea and teaching effect of the “processed” language promotion links are designed. Based on multiple rounds of reflective teaching practice, the principle of evaluation focus and the specific method of “promoting learning through evaluation” are determined [13–15]. In recent years, with the active international cooperation of the team, POA has gradually exerted an international influence and has been the subject of keynote reports in academic seminars many times.

**Figure 1:**
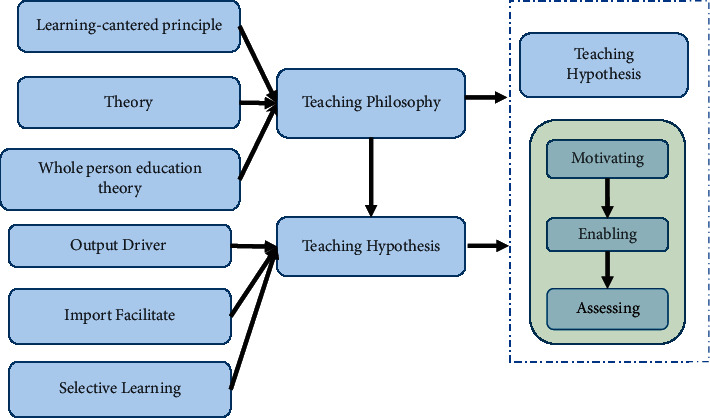
The theoretical system of POA.

POA is a new teaching theory proposed for middle and high-level foreign language learners to solve the problem of “separation of learning and application” in foreign language teaching and thus improve classroom efficiency [16]. POA takes knowledge output as the teaching goal and teachers' input learning and evaluation as an intermediary as a means to guide students to combine learning and use it to achieve the effect of optimizing the classroom [17]. The POA theoretical system is divided into three parts: teaching concept, hypothesis, and process. The teaching concept is the guiding ideology, the teaching hypothesis is the theoretical support, and the teaching process is a practical way. The three stages of the teaching process are intermediated by teachers, playing a leading, designing, supporting, and other roles [18]. The theoretical system of POA is shown in Figure 1.

### 2.2. BPNN

**Figure 2:**
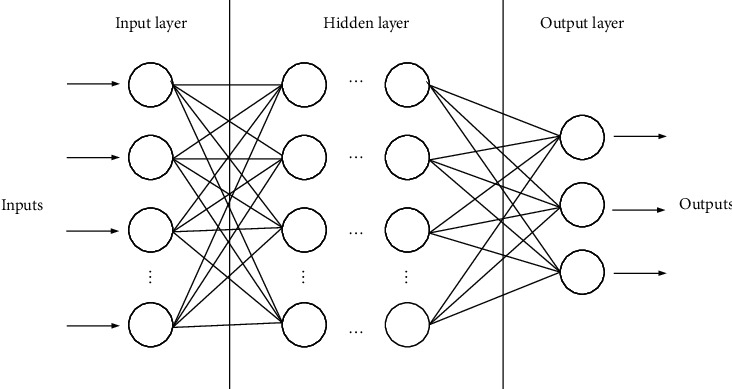
The three-layer BPNN structure.

BPNN is a typical representative of Artificial Neural Network (ANN) and is currently the most widely used ANN [19]. The BPNN is generated by simulating the neuron network (NN) structure of the human brain, which is a complex network composed of a large number of nodes connected to each other [20]. BPNN is a multilayer perceptron structure, which mainly contains three layers of input layer, hidden layer, and output layer [21]. The three-layer BPNN structure is shown in Figure 2.

The input layer and the output layer mainly store and transmit external information. All networks contain an input layer and an output layer. The main difference is that the number of hidden layers in the middle is different [22]. The hidden layer is not directly connected to the outside world, but its changes will have a direct impact on the relationship between the input layer and the output layer [23]. The BP algorithm includes two processes of signal forward and backpropagation in the process of learning. Forward propagation is carried out from the input layer to the output layer. If the error between the actual output signal and the expected output signal is too large, it needs to enter the backpropagation. Backpropagation is to propagate the output error layer by layer in the direction of the input layer through the hidden layer, distribute it to all units in each layer, and adjust the weight of each unit based on the error signal obtained by each layer. The connection strength and threshold between the input layer, the output layer, and the hidden layer enable the error to gradient descent. This process is repeated continuously until the error tends to the allowable range or reaches the presupposed practice frequency, and then the learning will be terminated [24].

**Figure 3:**
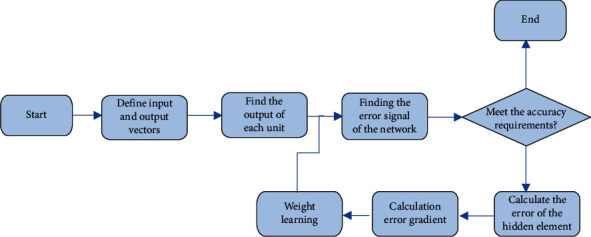
The process of BP algorithm.

The BP algorithm can be implemented through the following specific processes:

1. Select the appropriate input data and target data as the sample data for training the BPNN;
2. Establish a neural network model and initialize network parameters;
3. Through the constructed network training sample data, it is automatically completed by the computer program, and the cycle is continuously repeated until the preset value is reached;
4. Complete network training, determine weights and thresholds and output the results [25]. The specific process is shown in Figure 3.

In short, the BP algorithm can transform the signal input and output problems into nonlinear optimization problems. Combined with the gradient descent method, an iterative algorithm is used to solve the weights, and the added hidden layer nodes are used to increase the adjustable parameters of the optimization problem. The optimal solution can be obtained [26].

### 2.3. A Detection Method of English Reading Level Based on BPNN

**Figure 4:**
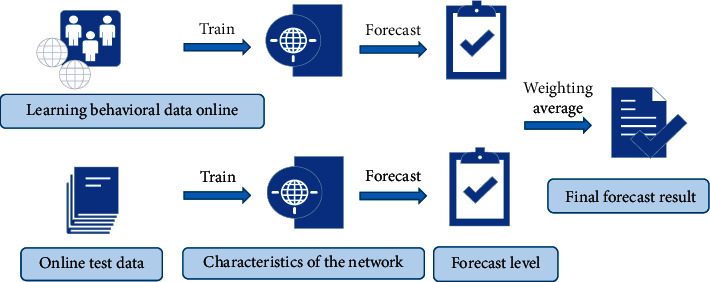
The overall design of the detection method of the English reading level based on BPNN.

A detection method of English reading level based on BPNN is designed, and it is used as one of the evaluation methods to carry out the teaching practice experiment integrating POA. The overall design of the method is shown in Figure 4.

The data is divided into two categories: student behavior data during online teaching and online reading test results. The former is subdivided into the downloads of courseware during online learning, video viewing, the number of operations during video viewing, the check-in situation of live interactive classrooms, classroom participation, etc. The latter is subdivided into classroom test results, random questions, and after-school online test results. The overall design of the detection method of the English reading level based on BPNN is shown in Figure 4. Firstly, the above two types of data are preprocessed, including noise removal and outlier detection. Secondly, two BPNNs are designed to process the characteristic data of learning behavior and of online reading test results. The “learning behavior feature network” and “online reading test results feature network” are constructed, and the neural network training and parameter tuning are carried out. Finally, the stability and robustness of the model are improved by the weighted average method [12].

Feature sequences need to be designed and characterized to be converted into numeric vectors that can be fed into the model interface [27]. For the characteristics of student behavior in online teaching, the Bag of Words (BoW) model is used, which does not consider the order and only uses the frequency of words or eigenvalues in sentences as the basis [28]. After the vectorization is completed, Term Frequency-Inverse Document Frequency (TF-IDF) technique is used to weigh the features to prevent overfitting [29]. TF-IDF can be expressed as shown in the following:

(1) $$\begin{array}{c} \text{TF}-\text{IDF}\left(l,i\;d\right)=\text{TF}{\left(l,i\;d\right)}^{*}\text{IDF}\left(l\right). \end{array}$$

TF(*l*, *i*  *d*) is the frequency of the behavior feature *l* in the student *i*  *d*. IDF(*l*) is the inverse document frequency, indicating the importance of the behavior feature *l*, and IDF(*l*) is shown in the following:

(2) $$\begin{array}{c} \text{IDF}\left(l\right)=\log\frac{N}{n_{l}+1}. \end{array}$$

*N* represents the total number of students, and *n*_*l*_ means the number of students with behavioral characteristic *l*. For the test data of the numerical class, the original data is scaled by a certain range mapping through normalized preprocessing [30].

To make the weights of the NN approximate to the real environment, the model initializes the weights and then multiplies them with random numbers and trains to obtain the approximate actual output of each layer of the network [31, 32].

(3) $$\begin{array}{c} x^{\left(i\right)}=f\left(s^{\left(i\right)}\right)=f\left(w^{\left(i\right)}x^{\left(i-1\right)}\right), \end{array}$$

**Figure 5:**
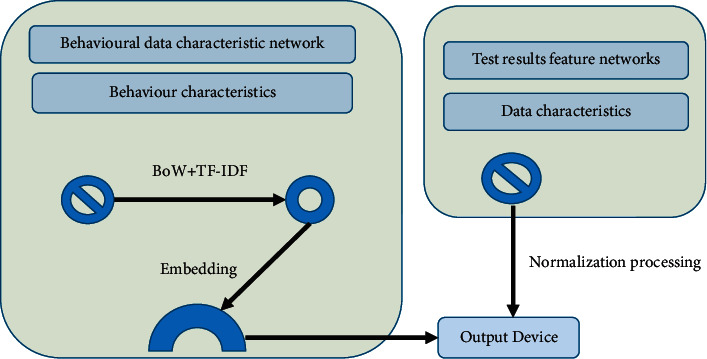
The output process of the model feature network.

*x*^(*i*)^ is the output value of the ith layer. *s*^(*i*)^ is the dot product value of the output of the *i* − 1-th layer and the weight. *f*(*s*^(*i*)^) represents the output value processed by the activation function, and *w*^(*i*)^ is the weight of the *i* -th layer. The output process of the model feature network is shown in Figure 5.

In Figure 5, the learned behavior features in the original features are firstly vectorized through Bow and TF-IDF, then trained through the BPNN, and finally connected to the output unit. The designed BPNN has 23 input nodes in one input layer. The research problem is mainly the prediction of continuous values. Therefore, the Relu activation function is used for the hidden layer, and the linear activation function can be used for the output layer. The same design approach is used in the reading test results feature network.

First, the training error *δ*_*j*_^(*i*)^ is calculated, as shown in the following:

(4) $$\begin{array}{c} \delta_{j}^{\left(i\right)}=f^{\prime }\left(s_{j}^{\left(i\right)}\right)\sum \delta_{k}^{\left(i+1\right)}w_{kj}^{\left(i+1\right)}. \end{array}$$

*δ*_*j*_^(*i*)^ is the training error of the *j*th neuron in the *i*th layer, and *w*_*kj*_^(*i*+1)^ expresses the connection weight value of the *k*th neuron in the *i*-th layer connecting to the *j*th neuron in the next layer.

Then, the weights are updated through backpropagation, and the correction of the weight value is shown in the following:

(5) $$\begin{array}{c} w_{ji}^{\left(i\right)}\left[k+1\right]=w_{ji}^{\left(i\right)}\left[k\right]+\eta \delta_{j}^{\left(n\right)}x_{i}^{\left(i-1\right)}. \end{array}$$

*η* is the learning rate.

The training objective function is shown in the following:

(6) $$\begin{array}{c} E=\sum \frac{1}{2}\sum {\left(d_{ij}-y_{ij}\right)}^{2}, \end{array}$$

*E* is the expected sum of errors in all training processes. *d*_*ij*_ is the expected output value of the *j*th neuron in the *i* th layer, and *y*_*ij*_ is the prediction error of the *j*th neuron in the *i*-th layer.

**Figure 6:**
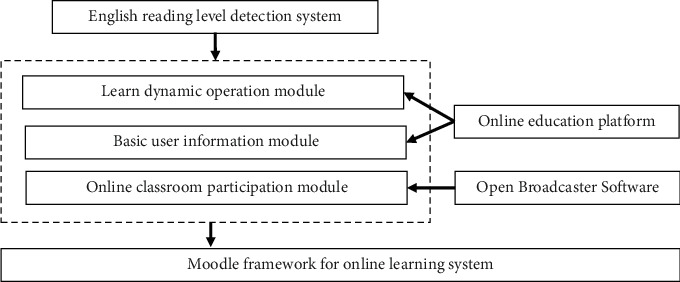
The structure diagram of the detection system of English reading level.

The widely used online learning system Moodle framework is selected as the development prototype. The detection system of English reading level can be divided into three parts: learning dynamic operation module, user basic information module, and online classroom participation module, among which the learning dynamic operation module is the core of the system [33]. The system obtains the predicted value close to the real result by constructing a complete operation chain, timely warns the students who are not in a good state, and optimizes the design of the subsequent courses. The structure diagram of the detection system of English reading level is shown in Figure 6.

### 2.4. The Practice of College English Reading Teaching with Integrated POA

**Figure 7:**
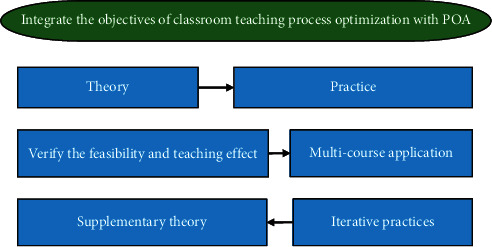
The objectives of optimizing the classroom teaching process with integrated POA.

It attempts to materialize POA into college English reading teaching. On the basis of the POA classroom teaching process, it refines and supplements specific content to optimize the teaching design. The objectives of optimizing the classroom teaching process with integrated POA are shown in Figure 7.

The specific practice methods of college English reading teaching are as follows:

  Teaching background: The effective duration of the planned teaching experiment is two semesters, 32 study weeks, 4 classes per week, and each class is 45 minutes. Due to the impact of the epidemic, online teaching is becoming more and more common, and most comprehensive universities have established their own teaching platforms or conducted live interactive teaching through software. To unify the experimental teaching form and control the variables, it takes online teaching as the practical teaching form, and the type of online teaching platform selected by teachers is not limited.
  Teaching content: Volume 3 and Volume 4 of the textbook “New Horizons College English” reading and writing course for college English.
  Research object: Non-English major sophomore students are taken as the research object. The students are divided into 2 A classes and 2 B classes based on the English score of 130 in the college entrance examination, with 30–35 students in each class. An experimental group and a control group are set up, and each group had a class *A* and a class *B*.
  Experimental design of POA teaching: The experimental group integrates POA method teaching, and the control group adopts traditional methods of teaching. To control the variables, the English reading courses of the two groups of students are taught by the same teacher, and the teaching is carried out according to the teaching plan uniformly formulated by the Teaching and Research Office of the School of Foreign Languages. There are three differences in the teaching of the two groups. One is that the experimental class in the guided course will specifically explain the POA teaching method. The second is that the experimental group needs to improve students' autonomy through the “driving” link. The third is that in addition to completing the chapter reading of the textbook, the experimental group also needs to carry out “promote” link learning to cultivate cross-cultural awareness. Besides, in the “promote” link, teachers choose appropriate auxiliary materials for the design of the study plan and upload them to the online teaching platform. In the “evaluation” link, the detection method of English reading proficiency based on BPNN designed above is used to evaluate the teaching results of two groups for two semesters. This part of the results can be used as a reference for optimizing the classroom process and has a learning promotion effect for students. Teachers make mutual evaluation forms, students conduct evaluations, and electronic files are made simultaneously. Teachers evaluate students' online classroom performance. Finally, the final evaluation results are formed by the evaluation results of students' mutual evaluation, teachers, and models in a ratio of 2 : 3 : 5.
  In the reading teaching activities carried out around the 10 units of the textbook, each unit has set reading ability improvement goals and humanistic reading appreciation goals. The experimental group completed a total of 8 reading output tasks every two weeks. To reduce the difficulty, students are gradually adapted to the POA teaching mode. The output tasks of each unit are divided into different small subtasks. The details of some reading tasks are shown in Table 1.
  The reading tasks are grouped by teachers in a heterogeneous way, and the teacher's tasks are mainly arranged online and specific requirements are put forward. Taking the sixth reading task as an example, it introduces the specific implementation process of English reading teaching that integrates POA.
  Driving link: First, the background of reading articles is built, and then students are guided to have a brief discussion and share to mobilize enthusiasm. Finally, the chapter articles are read, and the intragroup translation is carried out in the group as a unit. After that, the teacher will lead the reading and ask the students to try small-paragraph translation. After the chapter reading task is completed, an extracurricular reading training is carried out, and the reading task acceptance is carried out in the second reading training class.
  Facilitating link: 1 short video and 3 extracurricular reading articles are prepared by the theme of the chapter as input materials, and students are guided to accumulate content, language, and structure that can be used for output tasks. Firstly, 5 detailed questions are designed for each article of extracurricular reading to help students grasp the main body and keywords of the article. Secondly, 8 different western festival articles are provided for students, and students choose to do Jigsaw reading independently. Finally, a Power Point (PPT) is made and shared online in the form of a personal explanation [34]. Teachers know whether students have the ability to complete output tasks, promote “learning” by “evaluation,” and improve teaching efficiency. The rest of the students, as public judges, focused on sharing key points and learned relevant skills of public speaking.

### 2.5. Design of Experiment in Practice

**Figure 8:**
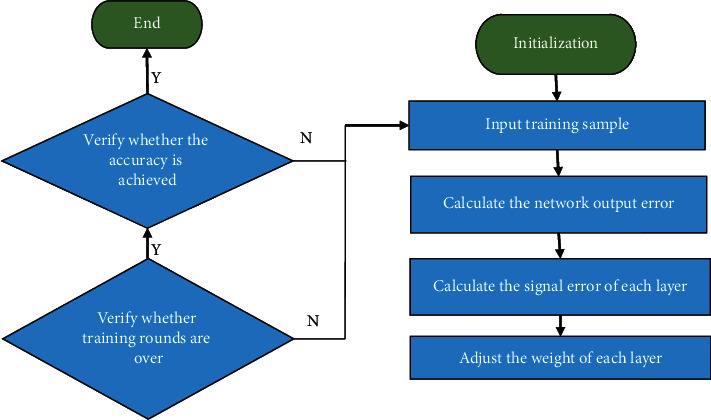
The model algorithm flow based on BPNN.

The postclass quiz and final grades are the most intuitive ways to reflect students' learning and knowledge in the whole semester. Therefore, the model verification indicator is based on the scores of the online postclass quiz and the reading module in the final English test. The data includes 4 readings of different difficulty levels. The article consists of 20 objective multiple-choice questions. The model test data set is identified with the student number as the serial number, and the multidimensional data is converted into structured data and preprocessed for model training. The model algorithm flow based on BPNN is shown in Figure 8.

The steps of model training are as follows:

(1) The model network weight *w*, bias term *b*, sample iteration times *q*, error *E*, learning rate *η*, and model training accuracy *E*_min_ are initialized, respectively [35]. After initialization, *w*, *b,* and *η* are random numbers. *η* is in the interval [0, 1]. *q* and *E* are 0, and *E*_min_ is a small positive number [36].
(2) The preprocessed raw data is input into the model to calculate the result. Simultaneously, the feature vector *L* and the real value *d* are initialized, and the output layer vector is calculated.
(3) The training data is set as *n* groups, and the model error is calculated. The error *E*^*k*^ of the model for the *k*th sample is shown in the following:
  (7) $$\begin{array}{c} E^{k}=\sqrt{\sum_{k=1}^{n}{\left(d_{k}-h_{k}\right)}^{2}}, \end{array}$$
*d*_*k*_ is the real value of the *k*th sample, and *h*_*k*_ is the output value of the *k*th sample in the output layer.
The total output error *E*_RME_ is shown in the following:
  (8) $$\begin{array}{c} E_{\text{RME}}=\sqrt{\frac{1}{n}\sum_{k=1}^{n}{\left(E^{k}\right)}^{2}}. \end{array}$$
Error signal of each layer is shown in the following equations:
  (9) $$\begin{array}{c} \sigma_{k}^{0}=\left(d_{k}-h_{k}\right)h_{k}\left(1-h_{k}\right), \end{array}$$
  (10) $$\begin{array}{c} \sigma_{j}^{y}=\sum_{k=1}^{\sigma }\sigma v_{jk}y_{j}\left(1-y_{j}\right). \end{array}$$
*v*_*jk*_ is the weight corresponding to the *j*th neuron in the *k*th layer of the hidden layer. *y*_*j*_ is the output value of the *j*th neuron in the hidden layer.
The weights of each layer are adjusted, as shown in the following equations:
  (11) $$\begin{array}{c} \Delta w_{jk}=-\eta \frac{\partial E}{\partial w_{jk}},\;j=1,2,\ldots ,n;k=1,2,\ldots ,m, \end{array}$$
  (12) $$\begin{array}{c} \Delta v_{jk}=-\eta \frac{\partial E}{\partial v_{jk}},\;j=1,2,\ldots ,n;k=1,2,\ldots ,m. \end{array}$$
*w*_*jk*_ is the weight corresponding to the jth neuron in the *k*th layer of the connection layer.
(4) Automatically check whether all training samples have been trained once. If the training result does not reach the preset accuracy after the training, the number of sample iterations *q* is incremented by 1, and the operation returns to step (2) to continue training. Verify whether the error of the result is within the accuracy requirement; if yes, the training is over; otherwise, *E* is initialized to 0, *q* is initialized to 1 and returns to step (2) for retraining.

Besides, the network parameters need to be optimized for the model, and 0.01 and 4000 are selected in combination with the training accuracy and the number of iterations. Before starting, the *w* and *b* between the input layer, hidden layer and output layer of the BPNN are initialized, and the random number is in the interval within [0, 1]. The number of nodes *N*_Hidden_ in the hidden layer is shown in the following equation:

(13) $$\begin{array}{c} N_{\text{Hidden}}=\frac{\left(N_{\text{Input}}+N_{\text{Output}}\right)}{2}. \end{array}$$

*N*_Input_ is the number of nodes in the input layer, and *N*_Output_ is the number of nodes in the output layer.*N*_Input_ = 23 and *N*_Output_ = 1 are set in experiment, so *N*_Hidden_ is 12. When *N*_Hidden_ is 12 and the number of training iterations is 4000, the training is carried out and the error is calculated.

The experimental results of the teaching practice take the results of students' mutual evaluation, teacher evaluation, and model evaluation as the final evaluation results in a ratio of 2 : 3 : 5. The independent sample *t*-test is analyzed for the data, and the evaluation results of the two groups of students are compared with the results before the experiment starts. The practical evaluation of the college English reading teaching mode integrating POA is carried out.

## 3. Results

### 3.1. Test Results of English Reading Level Model Using BPNN

**Figure 9:**
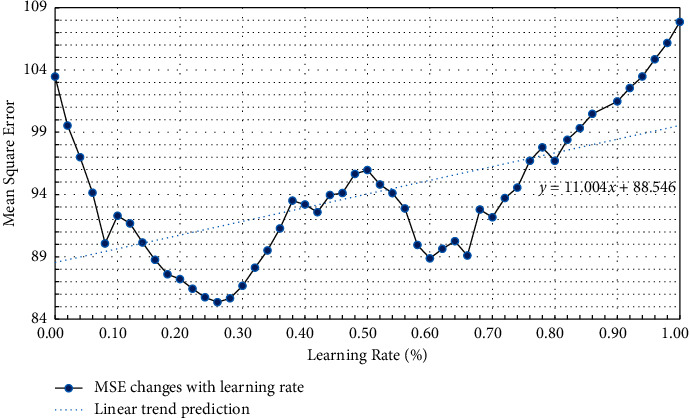
MSE changes with learning rate.

As the learning rate increases, the MSE of the model test is shown in Table 2 and Figure 9.

**Figure 10:**
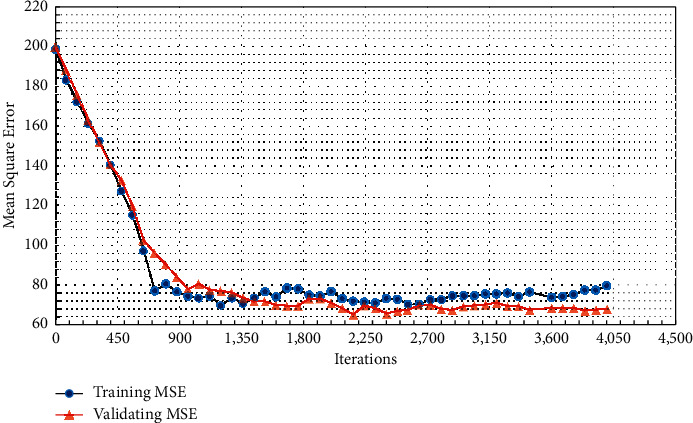
MSE changes with the number of training.

As the number of iterations increases, the MSE of the model test is shown in Table 3 and Figure 10.

Figure 9 indicates that when the learning rate is 0.26, the MSE of the model is the smallest. As the learning rate increases, the MSE shows a linear growth trend. Figure 10 expresses that as the number of training increases, the MSE of the model decreases. Before the number of training is 1000, the MSE of the model decreases exponentially. After the number of training is 1000, the reduction of the MSE tends to be stable, and overfitting occurs, so the optimal number of training is 1000.

### 3.2. Survey Results of Students Applying for College English Proficiency Test

**Figure 11:**
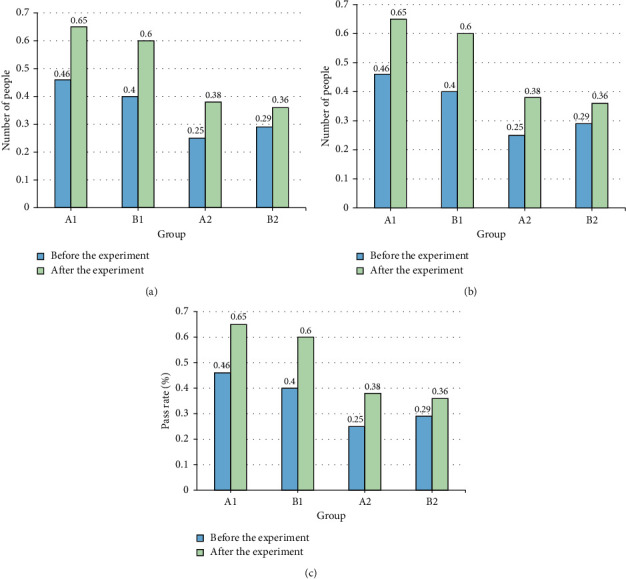
Survey results of students applying for college English proficiency test ((a) number of applicants; (b) number of actual exams; (c) pass rate).

Figure 11 shows the specific conditions of the experimental group and the control group before and after the start of the experiment for the CET-4 and CET-6 exams and their pass rates.

In Figure 11, *A*1 and *B*1 represent the experimental group; *A*2 and *B*2 represent the control group. Before and after the experiment, the number of people who signed up for the college English proficiency test, the number of actual exams, and the pass rate of the test group in the experimental group using the teaching method are higher than those in the control group. Moreover, the increase in the number of people in the experimental group is also higher than that in the control group after the experiment. It can be seen that the designed teaching method can effectively improve the enthusiasm of students to take the test and the pass rate.

### 3.3. The Results of College English Reading Teaching Practice Integrating POA

The independent sample *t*-test results of the comprehensive level of English reading in the experimental group and the control group before and after the experiment are shown in Tables 4 and 5.

Tables 4 and 5 show that the comprehensive English reading level of the two groups of students has improved after the experiment. Based on the total score of the comprehensive evaluation of 100 points, there is no significant difference between the average scores of the two groups before the experiment. However, the average scores of the two groups after the experiment, the gap is 9.88, of which the average score gap of class A is 6.21, and the gap of the average score of class B is 3.67. The comprehensive level of class A improves faster under the English reading teaching mode that integrates POA. The *p* value of the *t*-test of the results before the experiment is greater than 0.05, and there is no obvious difference in the comprehensive level of English reading between the two groups. The *p* value of the *t*-test of the test results after the experiment is less than 0.05, and there is an obvious difference in the English reading level between the two groups. Therefore, compared with the traditional teaching method, the teaching mode integrating POA is more effective in improving English reading ability. In the two A classes with a higher level before the experiment, the teacher's course progressed more smoothly, the completion of students' tasks is better, and the experimental effect is obvious. While in the two *B* classes with a lower level, due to the gap in individual English level, some students are not adapted to the teaching method of “combining learning with use,” the progress of the course is slow, and the completion of reading task of each chapter is low.

## 4. Conclusions

The traditional English reading teaching has serious test-taking thoughts, and the improvement of application ability is not paid attention. The teaching mode mainly focuses on the reading of articles in the classroom and exercises after class. Theoretical knowledge is separated from practice, focusing only on problems such as problem-solving skills. With the popularization of wireless networks and the improvement of online education, the college English reading teaching method integrating POA under the perspective of AI has broad application prospects. According to the teaching process of POA, the evaluation part is taken as a vital research module, the detection method of English reading level is designed by the BPNN, and the students' learning status and English reading level are comprehensively assessed. The theoretical practice of POA is integrated into the actual college English teaching. The experimental results indicate that the BP model has the smallest MSE when the learning rate is 0.26, which is 85.36, and the relationship between the MSE and the learning rate is linear growth. With the increase of the number of iterations, the MSE of the model gradually decreases, and the optimal number of training is 1000 times. The experimental results denote that before and after the experiment in the experimental group using the teaching method, the number of people who signed up for the college English proficiency test, the number of actual exams, and the pass rate of the test group are higher than those in the control group. Moreover, the increase in the number of people in the experimental group is also higher than that in the control group after the experiment. There is no significant difference in the test results of the two groups before the experiment, but the average test scores of the experimental group are obviously higher than those of the control group after the experiment. The *p* value of the *t*-test in the two groups is less than 0.05, indicating that there is a significant difference in the English reading level between the two groups after the experiment. Meanwhile, the difference between the results of class A before and after the experiment is larger than that of class B, showing that the teaching method integrating POA has a better teaching effect on students with higher English levels. Therefore, compared with the traditional teaching method, the teaching mode integrating POA is more effective in improving the English reading level of college students. Through the combination of AI and POA theory, the English reading teaching mode can be designed in the objective and multidimensional analysis results to evaluate the learning results during the teaching period, and simultaneously, it can accurately and quickly find problems in learning and teaching. The disadvantage is that the amount of data used is small, which makes the model still have certain errors. The feature data that the future model needs to accommodate and also needs to be expanded to make the final prediction structure closer to the true value. The teaching practice of POA theory also needs more long-term research and practice to adjust. It aims to transform POA theory into practice and provide a reference for college English reading teaching.

## Figures and Tables

**Table 1:** Topics for some reading tasks.

Assignment	Unit theme	Output task
1	Unit 1 curriculum selection	Group discussion: university course selection
2	Unit 2 children's aid society	Events to share: children foundation example
3	Unit 3 world famous painting	Painting appreciation: Van Gogh's self-portrait
4	Unit 4 eastern and western architecture	General debate: indoor office is better
5	Unit 6 courage	Press release reading: news report
6	Unit 8 eastern and western festivals	Passage-based reading: festival custom

**Table 2:** The variation of the MSE of the model test with the learning rate.

Learning rate (%)	Mean square error	Learning rate (%)	Mean square error
0.00	103.4606	0.50	95.9588
0.02	99.53603	0.52	94.80389
0.04	96.9959	0.54	94.10976
0.06	94.14807	0.56	92.87686
0.08	90.06871	0.58	89.95014
0.10	92.29888	0.60	88.87154
0.12	91.68075	0.62	89.63971
0.14	90.14138	0.64	90.25357
0.16	88.75418	0.66	89.09745
0.18	87.59866	0.68	92.78915
0.20	87.21192	0.70	92.1727
0.22	86.44132	0.72	93.71009
0.24	85.74764	0.74	94.55518
0.26	85.36181	0.76	96.70645
0.28	85.66798	0.78	97.78139
0.30	86.66707	0.80	96.70356
0.32	88.12709	0.82	98.39526
0.34	89.51064	0.84	99.31757
0.36	91.27759	0.86	100.471
0.38	93.50593	0.90	101.4699
0.40	93.19672	0.92	102.5458
0.42	92.57966	0.94	103.4679
0.44	93.96306	0.96	104.8518
0.46	94.11508	0.98	106.1589
0.48	95.65278	1.00	107.8498

**Table 3:** The variation of the MSE of the model test with the number of iterations.

Iterations	Training mean square error	Validating mean square error	Iterations	Training mean square error	Validating mean square error
0.00	198.671	199.5625	2000.00	76.61911	70.84998
80.00	183.1189	188.0069	2080.00	73.06961	68.18764
160.00	172.0078	175.5633	2160.00	71.74578	65.08949
240.00	161.3411	163.1215	2240.00	71.30307	69.53913
320.00	152.4548	151.5659	2320.00	70.86727	68.21358
400.00	140.4548	140.4548	2400.00	73.09468	65.55469
480.00	127.1232	132.4557	2480.00	72.65716	66.89667
560.00	115.1232	119.5711	2560.00	70.43926	67.35495
640.00	97.35236	102.2464	2640.00	70.44877	70.03632
720.00	76.91051	96.03286	2720.00	72.67964	70.04756
800.00	80.47038	90.25508	2800.00	72.69088	67.82966
880.00	76.48249	84.04842	2880.00	74.4773	67.39559
960.00	74.26459	77.83225	2960.00	74.48941	69.18115
1040.00	73.38694	80.50497	3040.00	74.50065	69.63511
1120.00	74.28361	77.40856	3120.00	75.39645	70.09252
1200.00	69.84695	76.97882	3200.00	75.41375	70.99524
1280.00	73.41029	76.09684	3280.00	75.87895	69.22784
1360.00	70.75054	73.44228	3360.00	74.11673	69.23735
1440.00	73.42758	71.67056	3440.00	76.35625	67.47255
1520.00	76.54821	71.6792	3600.00	73.7086	68.37181
1600.00	73.89883	69.91094	3680.00	74.16515	68.38565
1680.00	78.35625	69.47341	3760.00	75.06615	68.39256
1760.00	77.9265	69.48898	3840.00	77.30134	67.06961
1840.00	74.82404	73.05145	3920.00	77.31172	67.52616
1920.00	74.39083	73.0601	4000.00	79.55037	67.99568

**Table 4:** Independent sample *t*-test results of English reading comprehensive level before the experiment.

Group	Class	*n*	Mean	SD	*t*	*p*
Experimental group	*A*1	32	85.98	4.255	0.384	0.699
	*B*1	30	72.56	4.019		
Control group	*A*2	31	84.22	1.102		
	*B*2	32	73.43	1.267		

**Table 5:** Independent sample *t*-test results of English reading comprehensive level after the experiment.

Group	Class	*n*	Mean	SD	*t*	*p*
Experimental group	*A*1	32	92.37	5.684	1.345	0.032
	*B*1	30	78.33	3.712		
Control group	*A*2	31	86.16	2.524		
	*B*2	32	74.66	3.563		

## Data Availability

The data used to support the findings of this study are included within the article.
